# Successful radiofrequency catheter ablation of focal atrial tachycardia originating from right atrial appendage anomaly

**DOI:** 10.1016/j.hrcr.2024.03.001

**Published:** 2024-03-11

**Authors:** Reshma Amin, Brian Campbell, Michael Green, Ronak Rajani, Matthew Wright, John Whitaker

**Affiliations:** ∗ Guys’ and St. Thomas’ NHS Foundation Trust, London, United Kingdom; † School of Biomedical Engineering and Imaging Sciences, King’s College London, London, United Kingdom

**Keywords:** Atrial tachycardia, Tachycardia-induced cardiomyopathy, Radiofrequency ablation, Right atrial appendage anomaly, Intracardiac echocardiogram

**Key Teaching Points**

• Radiofrequency ablation within a pouch originating from an anomalous right atrial (RA) appendage for treatment of an incessant atrial tachycardia causing hemodynamic compromise and tachycardia-mediated cardiomyopathy is feasible and can be performed effectively and safely.
• Circumferential ablation of RA pouches to achieve electrical isolation can be trialed as an initial ablation strategy, particularly if high catheter impedance is recorded on manipulation within the structure; however, anatomical ridges may make this practically challenging.
• Catheter maneuverability within these spaces can be hindered by complex trabeculations and high impedance may be observed in some cases. Careful monitoring of impedance during ablation is mandatory when ablation within these structures is attempted.

## Introduction

Focal atrial tachycardia (AT) accounts for up to 10% of supraventricular tachycardias; however, despite this low incidence, their management presents difficulties owing to limited response to medical management including direct current cardioversion (DCCV), as well as a predisposition to initiate other atrial arrhythmias.^1^ ATs may result from microreentry, triggered activity, or enhanced automaticity and typically arise from characteristic locations in the atria, where the anatomic configuration may predispose to ectopic impulse generation and propagation.^2^ In approximately 10% of patients incessant AT may lead to tachycardia-induced cardiomyopathy (TCM). Tachycardia-mediated left ventricular (LV) systolic dysfunction often resolves following tachycardia termination,^3^ highlighting the importance of successful AT elimination in this cohort. The majority of AT can be successfully eliminated using radiofrequency (RF) catheter ablation and although success rates of acute arrhythmia suppression are reasonably high, the anatomical site of origin (SOO) has been demonstrated to be a limiting factor to arrhythmia suppression following a single procedure.^2^ This may be owing to thicker or inaccessible tissue rendering the arrhythmia focus difficult to reach with RF energy, or owing to proximity to a critical structure limiting safe delivery of RF energy. The RAA has been reported as a SOO of focal AT ^4^, and has been associated with difficulties in treating endocardially, due to thicker tissue and complex trabeculations. Successful strategies have included surgical excision of the right atrial appendage (RAA), isolation of the RAA, and focal ablation within the RAA.^4^, ^5^, ^6^ Right atrium (RA) pouches are an uncommon phenomenon that have been reported as a SOO of focal AT and are usually found in relation to the cavotricuspid isthmus (CTI), where they can complicate ablation of CTI-dependent atrial flutter.^7^ We present a case of focal AT complicated by severe LV systolic dysfunction, resulting in hemodynamic instability, in a patient in whom the AT SOO was an anatomical variant of an RAA, not previously described in the literature, with connections to the lateral RA and superior vena cava (SVC).

## Case report

**Figure 1:**
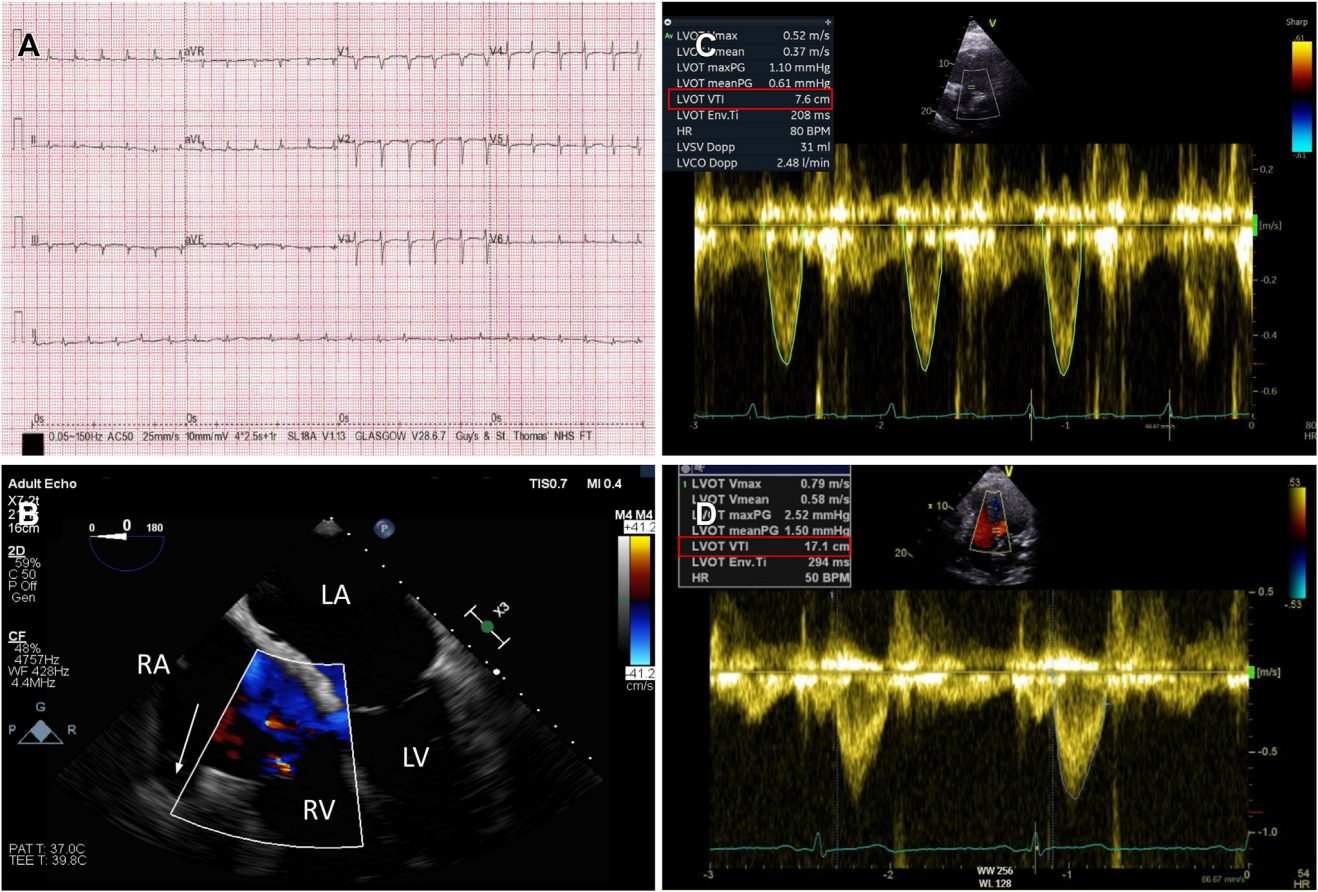
**A:** Twelve-lead electrocardiogram demonstrating AT with atrial cycle length of approximately 400 ms and negative p waves in V_1_. **B:** Transesophageal echocardiogram mid esophageal 0-degree view demonstrating abnormal cavity anterior to the RA free wall (*white arrow*). **C, D:** LVOT VTI on transthoracic echocardiogram increase, from prior to AT ablation with ongoing arrhythmia, to 4 months following AT ablation in sinus rhythm. AT = atrial tachycardia; RA = right atrium; LA = left atrium; RV = right ventricle; LV = left ventricle; LVOT VTI = left ventricular outflow tract velocity time integral.

A 58-year-old man presented to his local hospital with difficulty breathing and orthopnea. He had a recent history of coryzal illness and travel and no other past medical history. On clinical examination he was found to be tachycardiac and hypotensive (heart rate 180 beats/min, blood pressure 100/70 mm Hg) and a 12-lead electrocardiogram demonstrated a regular narrow complex tachycardia (Figure 1). This was treated sequentially with adenosine administration, resulting in transient atrioventricular block without interruption of the tachycardia; intravenous metoprolol, resulting in brief cardiorespiratory arrest leading to endotracheal intubation; and DCCV, which was unsuccessful. Transthoracic echocardiogram demonstrated biventricular systolic dysfunction and LV ejection fraction was estimated between 10% and 15%. He continued to deteriorate with rising venous lactate that peaked at 14 mmol/L and ongoing hypotension despite administration of norepinephrine and dobutamine. At this time the patient was referred to our center for consideration of hemodynamic support through venoarterial extracorporeal membranous oxygenation (VA-ECMO). Following assessment from the ECMO team, conventional retrieval was undertaken, and introduction of mechanical circulatory support was deferred. Medical therapy was attempted through the use of intravenous amiodarone and digoxin. Multiple attempts at DCCV were undertaken which did not interrupt the tachycardia. Dobutamine, noradrenaline, and milrinone were required to maintain a perfusing blood pressure. Invasive coronary angiogram revealed unobstructed coronary arteries. The decision was made, following Heart Team discussion, to proceed with electrophysiology study and ablation.

### Invasive mapping and ablation procedures

**Figure 2:**
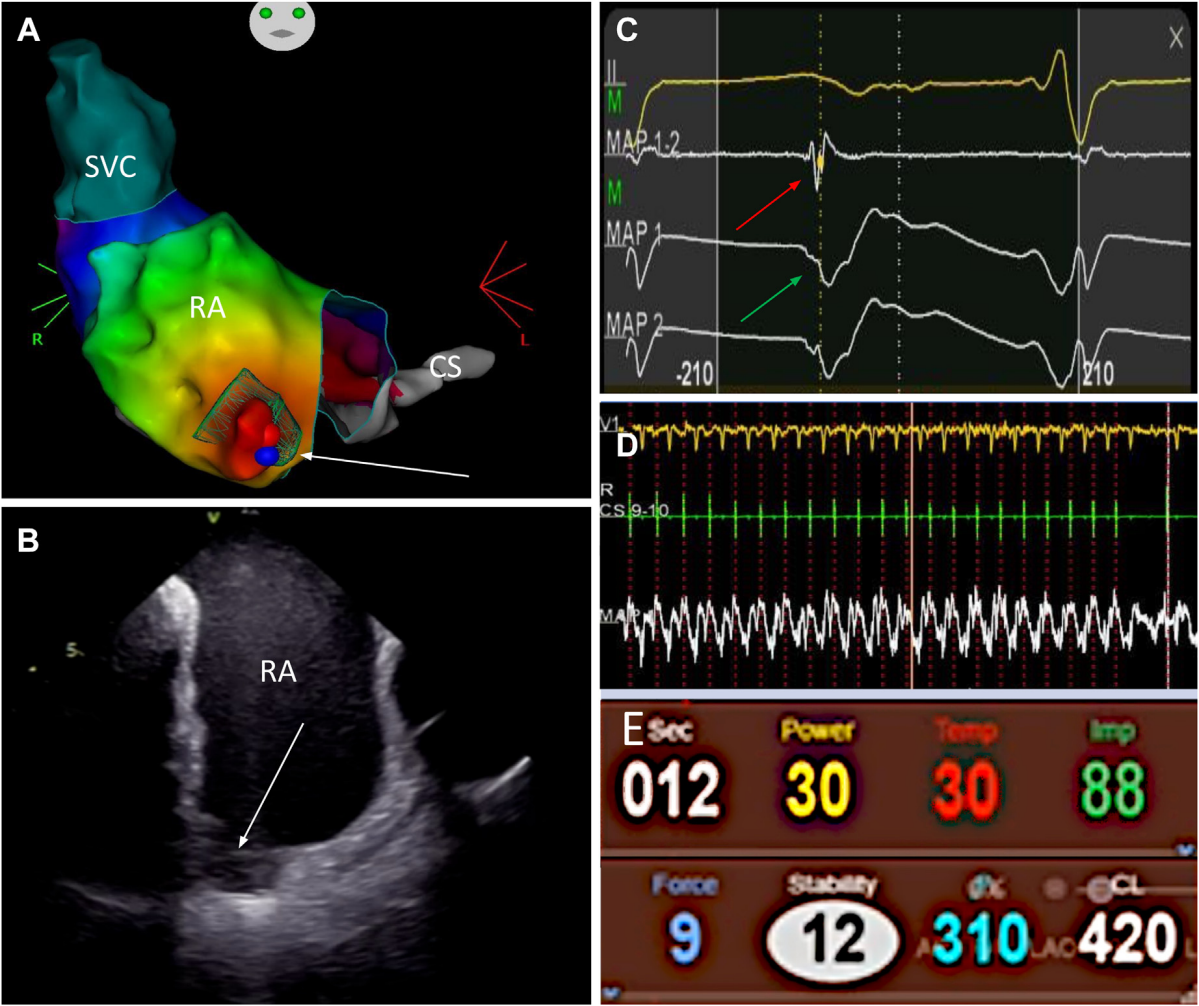
**A:** Electroanatomical mapping showing focal activation originating from aneurysmal pouch in the RA lateral wall with blue ablation lesion that resulted in final termination of tachycardia (*white arrow*). **B:** Intracardiac echocardiogram image of RA insertion of accessory lobe of appendage. **C:** Electrogram on ablation catheter at site of termination; red arrow indicates bipolar recording with activation early in p wave, green arrow indicates unipolar recording with QS morphology. **D:** Surface electrocardiogram V_1_ lead, CS catheter, and ablation demonstrating ablation onset and rapid cessation of tachycardia. **E:** Power and impedance during ablation within RA pouch. CS = coronary sinus; RA= right atrium; SVC = superior vena cava.

The patient was transferred to the cardiac catheter laboratory from the intensive therapy unit under general anesthesia. The procedure was performed with transesophageal echocardiogram (TEE) guidance via right femoral venous access, obtained under ultrasound guidance, using the CARTO® electrophysiology mapping system (Biosense Webster, Inc.; Irvine, CA) without fluoroscopy. TEE prior to procedure initiation noted a “windsock”-like pouch anterior to the RA free wall (Figure 1, Supplemental Video 1). Unfractionated heparin was administered to maintain activated clotting time >300 seconds, in accordance with institutional protocol. A DECANAV® mapping catheter (Biosense Webster, Inc.) was placed in the coronary sinus (CS). Heart rhythm at baseline was a narrow complex tachycardia with a tachycardia cycle length (TCL) of 400–460 ms with proximal-to-distal CS activation and 1:1 AV conduction. Activation mapping was undertaken using a PENTARAY® catheter (2-5-2 spacing; Biosense-Webster, Inc.), demonstrating a focal activation arising from an outpouching on the lateral wall of the RA (Figure 2A). Node-dependent SVT had already been excluded on the basis of the response to adenosine administration; therefore, a diagnosis of focal AT was made. AT entrainment was undertaken at the distal CS, at the proximal CS, at the ridge of the RA pouch, and within the RA pouch, yielding postpacing interval – total cycle length measurements of >300 ms, >200 ms, 100 ms, and 50 ms, respectively. Earliest bipolar signal activation and a QS unipolar signal were recorded within the RA pouch. Initial ablation strategy was at the site of earliest atrial local activation at power of 40–50 watts with contact force between 10 and 20 grams, resulting in initial impedance drop of 10 ohms targeting an ablation index of 450–550. Starting impedance within the pouch was 100 ohms, with reduction to 90 ohms following ablation, which resulted in slowing of the AT cycle length without termination. Following several further unsuccessful attempts to ablate at the earliest point of activation, an attempt was made to electrically isolate the pouch with circumferential ablation; however, a medial ridge at the base of the outpouching made stability and contact difficult and isolation was unsuccessful. Further ablation was therefore undertaken, with greater force, within the RA pouch. This terminated tachycardia, with no arrhythmia recurrence following a 20-minute waiting time. The phrenic nerve was localized with high-output pacing to the posterolateral portion of the RA and confirmed to be remote from all the ablation sites.

**Figure 3:**
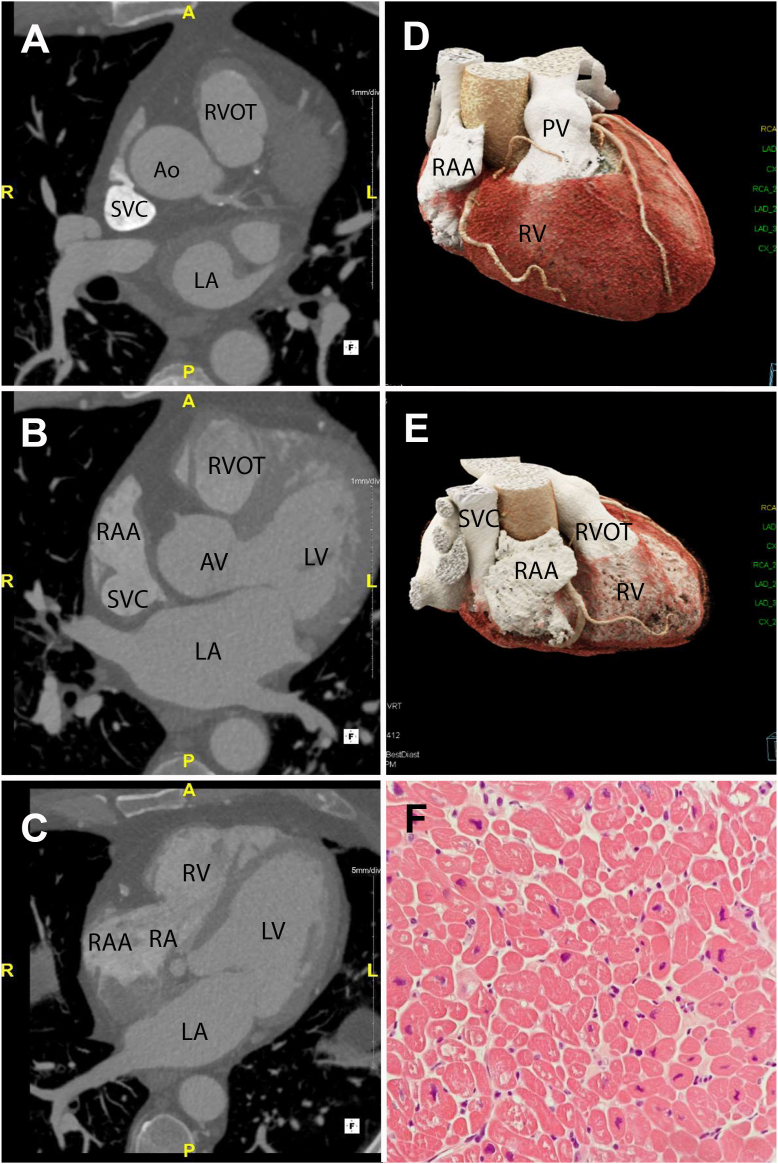
**A:** Axial slice of cardiac computed tomography angiography showing small contrast-filled channel arising from a superior aspect of the SVC. **B:** Anomalous RAA arising from SVC just superior to the junction of the SVC to the RA. **C:** Connection of RAA to RA; note in panels B and C the absence of the conventionally sited RAA. **D, E:** Three-dimensional cinematic volume-rendered images showing oblique views of the right heart and location of anomalous RAA. **F:** Histology of LV biopsy demonstrating occasional hypertrophic myocytes with no inflammation or fibrosis. Ao = aorta; LA = left atrium; LV = left ventricle; RA = right atrium; RAA = right atrial appendage; RV = right ventricle; RVOT = right ventricular outflow tract; SVC = superior vena cava.

Within 24 hours there was recurrence of AT with associated hemodynamic compromise; therefore, a second procedure was undertaken under general anesthesia and intracardiac echocardiogram (ICE) guidance. ICE was used to carefully delineate the RA outpouching and its ostium (Figure 2B). TCL was 440–530 ms, again with proximal-to-distal CS activation. Electroanatomical mapping was undertaken, with earliest atrial activation confirmed at the inferolateral aspect of the RA pouch, in close proximity to the previous ablation site, which had resulted in termination of tachycardia; however, on this occasion the site of earliest activation electrogram was slightly more inferior in the pouch. Anatomic delineation of the pouch with the use of integrated ICE imaging was valuable for successful navigation into the pouch and to the site of earliest activation. Ablation within the RA pouch at this site was delivered at 30 watts and increased incrementally to 50 watts with contact force of 10–20 g and undertaken with close impedance monitoring. The AT accelerated with delivery of energy, then slowed and terminated within 12 seconds of onset of ablation (Figure 2C–2E). There was no recurrence following a 20-minute waiting time, and burst pacing within the RA pouch until the atrial tissue was refractory did not reinduce arrhythmia. The patient was transferred back to the intensive therapy unit, where hemodynamic support was weaned and eventually discontinued. At 4-month follow-up he has maintained sinus rhythm without arrhythmia recurrence. Repeat transthoracic echocardiography showed an improvement in LV systolic function to 40%–45% (Supplemental Video 1). To further delineate anatomy following the findings on periprocedural TEE, an electrocardiogram-gated cardiac computed tomography was performed using a dedicated triphasic contrast protocol to enable left- and right-sided heart chamber evaluation. This showed an absence of an RAA arising from the anterior and superior aspect of the RA. Note was made, however, of a small contrast-filled channel arising from the SVC and a further larger contrast-filled cavity arising from the SVC just superior to the juncture with the RA. This cavity had the typical appearance of an RAA with pectinate muscle and coursed inferiorly and medially to connect with the RA just lateral to the right-sided atrioventricular groove (Figure 3A–3E). The lateral insertion of this accessory lobe of the RAA corresponds to the outpouching that was delineated on electroanatomical mapping and ICE imaging. Biopsy of the LV septum showed no significant fibrosis or evidence of inflammation, granulomata, iron deposition, or amyloid (Figure 3F).

## Discussion

Focal AT can be defined by atrial activation originating from a discrete location of atrial myocardium, arbitrarily defined as less than 2 cm in size, with centrifugal spread. The anatomical structures responsible for the origins of focal AT are clustered and in the RA are found most commonly in the crista terminalis (CT), tricuspid annulus, CS ostium, para-Hisian region, and, more rarely, the RAA.^8^ These sites are theorized to have a predisposition to AT, as they encompass a junction of histological tissue or area of marked tissue anisotropy, which may predispose to microreentry as well as promote propagation of ectopic impulses.

Focal AT may arise owing to abnormal automaticity, triggered beats, or microreentry. Triggered activity is defined as impulse initiation that is caused by myocyte calcium flux during phase 4 of the cardiac action potential, resulting in an increase in the myocyte membrane potential. If this afterdepolarization is of sufficient magnitude to reach the threshold voltage, another action potential is triggered; and if this is propagated, a premature atrial complex occurs.^9^^,^^10^ There is currently no strategy to definitively assign a mechanism to an AT that appears focal^11^; however, some investigators have used a variety of characteristics, including mode of initiation, presence of delayed afterdepolarizations at the site of origin, response to medications, and response to overdrive pacing, to attempt to systematically classify ATs according to mechanism,^12^ and there is compelling evidence supporting a role for each of these in different tachycardias.^11^ In this case, the tachycardia was incessant and therefore initiation and response to medications was not determined. Monophasic action potentials were not assessed during the clinical case, so the presence of delayed afterdepolarizations was not assessed. We note that at the site of successful ablation, overdrive pacing yielded a return cycle length of 50 ms above the TCL, which would appear to favor abnormal automaticity or triggered activity as a mechanism. Differences in fiber orientation where trabeculations of the pectinate muscles within the RAA meet the smooth-walled right atrial body at the CT,^13^ as well as pronounced gradients in tissue thickness,^14^ give rise to marked anisotropy in this region, as well as significant source-sink mismatches. These characteristics may facilitate abnormal impulse generation as well as propagation, a phenomenon that is likely to contribute to the observed preferential localization of focal AT to areas with these characteristics, such as the CT. We hypothesize that the same anatomic characteristics at the SOO in this case served as substrate characteristics that may have promoted abnormal automaticity or triggered activity, and in either case would be expected to promote impulse propagation and allowed the abnormal impulse to activate the atrium.

The conventional approach to ablation of focal AT, centered on identification of the origin of activation via intracardiac mapping and subsequent ablation at the site of a QS unipolar recording, has been highly predictive of successful outcomes both acutely and with regard to arrhythmia recurrence.^12^ Hemodynamically unstable patients with incessant tachycardia and TCM still present a challenge, particularly those with AT originating from an anatomical abnormality. To our knowledge this is the first documented case of successful RF ablation of a focal AT originating from an accessory lobe of an anomalous RAA. The RAA is an uncommon SOO for focal AT, being implicated in 3.8%–8%^6^^,^^15^; however, these ATs have some identifying features similar to this case despite the anomalous anatomy, such as a negative p wave in V_1_ becoming progressively more positive across the precordial leads, the incessant nature of the arrythmia, and successful ablation at the site of earliest endocardial activity.^6^^,^^15^ The anatomical variant of an atrial pouch is rare. There have been documented focal ATs originating from the tip of a diverticulum in the left atrium: in 1 case the diverticula were located adjacent to the right superior pulmonary vein; in the other, 2 pouches were observed, 1 anterior to the right superior pulmonary vein and the other a high septal RA pouch. In all cases, high impedance was recorded when manipulating catheters within the pouches, which restricted delivery of RF at the tip or SOO. The ablation strategy therefore was circumferential ablation around the structures.^16^ This circumferential approach was also the preferred initial strategy in our case owing to the first trial of ablation within the pouch resulting in slowing of the tachycardia but not termination. Extensive ablation within the pouch as the initial approach was considered higher risk owing to the potential for perforation. The anatomical ridge at the pouch ostia hindered stability of ablation and therefore prevented isolation via circumferential ablation. Direct ablation at the source was therefore the only remaining option and, in this hemodynamically unstable patient with presumed TCM, facilitating AT termination was of paramount importance. In an atrial pouch the myocardium tends to be thinner, and direct ablation within can be challenging owing to poor blood flow resulting in suboptimal power delivery, resulting in rapid temperature and impedance increase. An ablation strategy for atrial pouches within the region of the CTI has been proposed, using irrigated catheters with measured up-titration of energy delivery to avoid sudden impedance rise—a strategy that can be translated to ablation within pouches located in other anatomical locations within the RA,^7^ as was performed in this case, resulting in successful termination at the SOO. In this case the outpouching has subsequently been demonstrated to be likely due to an anomalous lobe of the RAA, which may explain the relatively consistent impedance measurements in this structure. This unusual anatomic variant emphasizes the importance of careful anatomic delineation of the anatomic basis for AT, which may be readily achieved intraprocedurally through the use of ICE.

## Conclusion

We report the first case in documented literature of focal AT arising from an accessory lobe of the RAA inserting anterior to the free wall of the RA with direct RF ablation within this structure, resulting in successful termination of focal AT. In this case as alternative ablation strategies were unsuccessful, namely circumferential ablation, the decision was made to attempt ablation within the structure, even before its delineation with cross-sectional imaging, as the patient was in refractory heart failure. Mapping of the region demonstrated catheter impedance within the pouch consistent with the rest of the RA, and during ablation appropriate temperature and impedance drop were achieved, suggesting adequate blood flow within the structure. Our case illustrates the need for alternative, potentially higher-risk ablation strategies when unusual anatomical sources are found to be the focal origins of arrhythmia and the value that ICE adds in the intraprocedural delineation of unusual anatomies. In this case the ablation within the pouch did not result in an adverse outcome; however, accounting for the thin wall of an aneurysmal structure in comparison to atrial tissue, the potential risk for perforation is higher and this risk must be considered against the clinical benefit.

## Disclosures

The authors have no conflicts of interest to disclose.

## Appendix Supplementary Data

Supplemental Video 1 Cine loop of echocardiogram in parasternal short axis view showing severe left ventricular (LV) systolic function in Atrial Tachycardia and subsequent improvement in LV systolic function once sinus rhythm has been restored, following catheter ablation.
